# *LRRK2* Attenuates Antioxidant Response in Familial Parkinson’s Disease Derived Neural Stem Cells

**DOI:** 10.3390/cells12212550

**Published:** 2023-10-31

**Authors:** Jeffrey Kim, Etienne W. Daadi, Elyas Sebastien Daadi, Thomas Oh, Michela Deleidi, Marcel M. Daadi

**Affiliations:** 1 Southwest National Primate Research Center, Texas Biomedical Research Institute, San Antonio, TX 78227, USA; 2 Department of Cell Systems & Anatomy, San Antonio, TX 78229, USA; 3 Institut Imagine, INSERM UMR1163, Paris Cité University, 75015 Paris, France; 4 Department of Radiology, Long School of Medicine, University of Texas Health at San Antonio, 7703 Floyd Curl Dr., San Antonio, TX 78229, USA

**Keywords:** induced pluripotent stem cells, neural stem cells, single cell transcriptomics, target identification, Parkinson’s disease

## Abstract

Parkinson’s disease (PD) is the second most prevalent neurodegenerative disease, characterized by the loss of midbrain dopaminergic neurons which leads to impaired motor and cognitive functions. PD is predominantly an idiopathic disease; however, about 5% of cases are linked to hereditary mutations. The most common mutation in both familial and sporadic PD is the G2019S mutation of *leucine-rich repeat kinase 2 (LRRK2)*. Currently, it is not fully understood how this mutation leads to PD pathology. In this study, we isolated self-renewable, multipotent neural stem cells (NSCs) from induced pluripotent stem cells (iPSCs) harboring the G2019S *LRRK2* mutation and compared them with their isogenic gene corrected counterparts using single-cell RNA-sequencing. Unbiased single-cell transcriptomic analysis revealed perturbations in many canonical pathways, specifically *NRF2*-mediated oxidative stress response, and glutathione redox reactions. Through various functional assays, we observed that G2019S iPSCs and NSCs exhibit increased basal levels of reactive oxygen species (ROS). We demonstrated that mutant cells show significant increase in the expression for KEAP1 and decrease in NRF2 associated with a reduced antioxidant response. The decreased viability of mutant NSCs in the H_2_O_2_-induced oxidative stress assay was rescued by two potent antioxidant drugs, PrC-210 at concentrations of 500 µM and 1 mM and Edaravone at concentrations 50 µM and 100 µM. Our data suggest that the hyperactive LRRK2 G2019S kinase activity leads to increase in KEAP1, which binds NRF2 and leads to its degradation, reduction in the antioxidant response, increased ROS, mitochondria dysfunction and cell death observed in the PD phenotype.

## 1. Introduction

Parkinson’s disease (PD) is a neurodegenerative disorder characterized by the progressive degeneration of the midbrain dopaminergic system and other neural systems and organs [1,2]. Symptoms of PD include tremors, rigidity, bradykinesia, akinesia, and other non-motor symptoms including cognitive impairment, depression, and dementia [3,4,5,6,7]. Even though PD is considered an idiopathic disease, at least 10% are family linked cases [8]. One of the most common genetic mutations found in familial PD was identified in the *leucine-rich repeat kinase 2 (LRRK2)* gene [9,10,11,12]. LRRK2 exhibits kinase and GTPase activity with functions linked to transcription, translation, autophagy, mitochondrial function, and vesicular transport [13,14,15,16,17,18]. The most common *LRRK2* mutation is the G2019S point mutation located within the kinase domain [9,12]. High prevalence is observed in Ashkenazi Jewish patients and North African Berber and Arab patients [19,20,21] while less common in Asian patients [22,23,24,25,26,27]. This particular mutation leads to hyper-kinase activity of LRRK2 [28,29,30,31] with a disease phenotype comparable to idiopathic PD [32,33]. However, currently it is not fully understood how this mutation causes PD [34] and how it impacts the transcriptional profile of neural cells.

Induced pluripotent stem cell (iPSCs) are a powerful tool for modeling genetic disorders as mutations are carried over during differentiation [35,36]. Mutant cells can be compared with an isogenic gene corrected cell line generated through gene editing of G2019S mutation to wild type, reducing LRRK2 activity and alleviating aberrations. In this study, we generated self-renewable neural stem cells (NSCs) from iPSCs harboring the G2019S *LRRK2* mutation and their isogenic gene corrected counterpart. We performed single-cell RNA-sequencing of NSCs and found significant differentially expressed genes involved in oxidative phosphorylation and antioxidant response. We observed that the G2019S *LRRK2* mutation contributes to increased oxidative stress due to a perturbed antioxidant response.

## 2. Materials and Methods

### 2.1. Induced Pluripotent Stem Cell (iPSC) Cultures

We previously reported in detail the derivation of IPSCs-*LRRK2* [37]. Fibroblasts of a 52-year-old PD male patient (ND29802, RRID:CVCL_DD50) with heterozygous G2019S *LRRK2* mutation were procured from the NINDS Repository at the Coriell Institute for Medical Research (Camden, NJ, USA). A frozen vial of the patient’s fibroblasts was thawed in 9 mL of fibroblast media (10% Fetal Bovine Serum/DMEM high glucose) and centrifuged at 1000 rpm 5 min at room temperature. The fibroblasts were re-suspended in fresh media, cultured and then expanded for 4 passages. A stock was frozen and 1 was transfected with the reprogramming factors. We used the non-integrative episomal vector system, which employs 4 episomal vectors: pCXLE-hOCT3/4-shp53, pCXLE-hSK, pCXLE-hUL, and pCXWB-EBNA1 (Addgene, Cambridge, MA, USA). The vectors were amplified in bacterial culture and purified using the Miniprep kit (Qiagen, Hilden, Germany) according to the manufacturer’s instructions. The advantage of EBNA1-based episomal reprogramming system is the use of non-integrating vectors with stable extrachromosomal replication. The vector and reprogramming gene sequences are cleared from the cells as the iPSC colonies are expanded. Eighty-two microliters of NHDF Nucleofector solution were mixed with 18 µL Supplement, 0.83 µg pCXLE-hOCT3/4-shp53, 0.83 µg pCXLE-hSK, 0.83 µg pCXLE-hUL, and 0.5 µg pCXWB-EBNA1. The fibroblasts were dissociated into single cells and collected by centrifuge 1000 rpm for 5 min at room temperature. The pellet was resuspended with the DNA-nucleofection mixture and applied to the U-023 program on the Nucleofector 2b device.

We also used the mutant *LRRK2* iPSC lines L1-1 and their isogenic gene corrected control L1-1GC edited using zinc finger nucleases (ZFNs) [38].

iPSCs were grown under feeder-free conditions using Geltrex Basement Membrane Matrix (Thermo Scientific, Waltham, MA, USA) supplemented with mTeSR Plus (Stem Cell Technologies, Vancouver, BC, Canada) stem cell media. The iPSCs were maintained with mTeSR plus media replaced daily. Cells were passaged every 4 days using 500 μM EDTA to non-enzymatically dissociate colonies from the Geltrex (Thermo Scientific, Waltham, MA, USA). Using immunocytochemistry (ICC) the pluripotency of the iPSCs were assessed for the expression of stem cell markers Oct4, NANOG, TRA-1-60 and SSEA4.

To correct the G2019S *LRRK2* mutation, iPSCs were transfected with 2 μg of each ZFNs construct, as well as 2 μg linearized targeting vector, using Amaxa Nucleofector (Lonza, Rockville, MD, USA), Nucleofection Solution for human stem cells I (Lonza, Rockville, MD, USA), according to the manufacturer’s instructions. The transfected cells were replated onto Matrigel (BD)-coated plates in the presence of medium previously exposed to MEFs and supplemented with 10 μM ROCK inhibitor (Ascent Scientific, Princeton, NJ, USA) and 5 ng/mL FGF2. A total of 50 μg/mL G418 (PAA) and 2 μM ganciclovir (Sigma, St. Louis, MO, USA) were used to select for homologous recombination. Resistant colonies were picked and clonally expanded on MEFs. Further perpetuation of these lines was carried out under feeder free conditions using Geltrex Basement Membrane Matrix (Thermo Scientific) and supplemented with mTeSR Plus (Stem Cell Technologies) stem cell media.

### 2.2. Isolation of NSCs from iPSCs

Self-renewable multipotent NSCs were derived from iPSCs as we previously reported [39] using NN1 media (NeoNeuron, San Antonio, TX, USA). Eighty percent confluent iPSC colonies were dissociated from Geltrex with 500 μM EDTA and re-suspended in NSC media composed of NN1 (NeoNeuron), 20 ng/mL bFGF (Stemgent, Beltsville, MD, USA), and 20 ng/mL EGF (EMD Millipore, Burlington, MA, USA). After seven days in culture, the cell suspensions were collected via centrifugation, single cell dissociated using StemPro Accutase (Thermo Fisher Scientific), re-suspended in fresh NN1 NSC culture media and plated in T-75 cell culture flasks (Corning, Corning, NY, USA) for expansion.

### 2.3. Differentiation of NSCs into Dopaminergic Neurons

NSCs grown as neurospheres were collected from T-75 cell culture flasks and plated onto Geltrex coated coverslips supplemented with NN1 media and the dopamine neuron differentiation factor DIF1 (NeoNeuron) as we previously reported [40]. Media was changed daily for 7 days before coverslips were fixed with 4% paraformaldehyde and processed for immunocytochemistry.

### 2.4. Drug Testing Assay Compounds

A 50 mM stock solution 7.2 pH of PrC-210 (Obvia Pharmaceuticals, Madison, WI, USA) was made in NN1 media. A stock of 10 mM of Edaravone (Sigma) was also made in NN1 media. NSCs were plated onto Geltrex coated coverslips each in 1% FBS NN1 media. They were spontaneously differentiated for 3 days in vitro (DIV). On day 3, the media was changed to 1% FBS NN1 and 200 μM H_2_O_2_ and concentrations 100 μM, 500 μM, or 1mM of PrC-210 or 50 μM to 100 μM of Edaravone for 2 h. After two hours, the cells were washed and replenished with fresh NN1 media. After 24 h, the coverslips were fixed and immunostained with multiple antibodies. Coverslips were imaged using the Zeiss LSM-800 Confocal microscope (Zeiss, Oberkochen, Germany).

### 2.5. Western Blotting

Protein was extracted from iPSCs and NSCs with RIPA buffer (Thermo Scientific) supplemented with 1:100 Halt protease and phosphatase inhibitor cocktail (Thermo Scientific). Protein concentration was measured using Pierce BCA protein assay (Thermo Scientific). Protein lysates were mixed with Laemmli sample buffer (Bio-Rad Laboratories, Hercules, CA, USA) and denatured at 95–100 °C for 5 min. SDS-PAGE was conducted using 4–20% Mini-PROTEAN TGX precast gels (Bio-Rad Laboratories). Protein was transferred to activated PVDF membranes (Bio-Rad Laboratories) using the Trans-Blot Turbo system (Bio-Rad Laboratories). Membranes were blocked using 5% non-fat dry milk in Tris-Buffered Saline (TBS) and 0.1% Tween for 1 h. The blots were incubated overnight at 4 °C in primary antibodies pT73 RAB10 (1:1000, Abcam, Waltham, MA, USA), RAB10 (1:1000, Abcam), KEAP1 (1:1000), NRF2 (1:1000), and β-actin (1:1000, Cell Signaling Technologies, Danvers, MA, USA). Afterwards, the blots were washed and incubated with secondary antibody anti-mouse HRP (1:2000, Bio-Rad Laboratories) or anti-rabbit HRP (1:2000, Bio-Rad Laboratories) for 1 h at room temperature. After the blots were washed, they were incubated in SuperSignal West Pico PLUS chemiluminescent (Thermo Scientific) substrate and imaged using UVP ChemStudio (Analytik-Jena, Upland, CA, USA). Mean intensity density was measured using VisionWorks Software (Analytik-Jena).

### 2.6. Gene Expression Analysis

RNA from iPSCs and NSCs were isolated using RNeasy-Plus Mini-kit (QIAGEN) according to the manufacturer’s protocol. cDNAs were synthesized from 1 μg of RNA using SuperScript IV First-Strand Synthesis kit (Invitrogen, Waltham, MA, USA), according to the manufacturer’s protocol. Gene expression analysis was conducted using PowerUp SYBR Green Master Mix (Thermo Scientific) using the StepOnePlus platform (Applied Biosystems, Foster City, CA, USA). Gene expression levels were calculated using the 2^−ΔΔCT^ method. The qPCR experiments were conducted in triplicate. The following primers were used for each target gene, the forward primer is listed first followed by the reverse primer: NRF2: GAGAGCCCAGTCTTCATTGC, TGCTCAATGTCCTGTTGCAT. KEAP1: AACTTCGCTGAGCAGATTGG, CGTAGAACCGTCGCTGTT. SOD2: AGGATCCACTGCAAGGAACA, GTGCTCCCACACATCAATCC. PRDX1: CATTCCTTTGGTATCAGACCCG, CCCTGAACGAGATGCCTTCAT. PRDX5: AGAAGGGTGTGCTGTTTGG, TCATTAACACTCAGACAGGCC. GPX1: TTCCCGTGCAACCAGTTTG, TTCACCTCGCACTTCTCGAA. GAPDH: AGAAAGTAGGGCCCGGCTAC, GGAGGCTGCGGGCTCAAT. GSTO1: ATTGATTACCTCATCTGGCCC, TCAGTTTTGGAGTGTGGTCTAC. GSTO2: CAGGTCTCCTTACCCATGTTTC, TAAACCACAGGGCAGTCAAG. GCLC: TGGATGCCATGGGATTTGGAA, CTCAGATATACTGCAGGCTTGGAA. GSS: AGCGTGCCATAGAGAATGAG, ATCCCGGAAGTAAACCACAG. TXN: GATGGTGAAGCAGATCGAGAG, CACGTGGCTGAGAAGTCAA. TXNRD1: GCATCCCTGGTGACAAAGAA, CCAACAACCAGGGTCTTACC.

### 2.7. Immunocytochemistry

Whole neurospheres were collected and plated on Geltrex coated coverslips for 24 h supplemented with NN1 NSC culture media. Immunostaining was performed as we previously described, the coverslips were fixed in 4% paraformaldehyde for 20 min and washed for 5 min with phosphate buffered saline (PBS) three times. Primary antibody solutions were made with PBS, 0.03% Triton X and 10% normal donkey serum. Triton X was excluded for cell surface proteins. Coverslips were incubated at 4 °C overnight with primary antibodies: NANOG (1:100, mouse, EMD Millipore), OCT4 (1:100, rabbit, EMD Millipore), SSEA4 (1:100, mouse, Developmental Studies Hybridoma Bank), TRA-1-60 (1:100, mouse IgM, Sigma-Aldrich, St. Louis, MO, USA), β-tubulin Class-III (1:100, mouse, Sigma-Aldrich), tyrosine hydroxylase (TH, 1:100, Pel-Freez), Nestin (1:100, rabbit, EMD Millipore), SOX2 (1:100, rabbit, Abcam), Vimentin (1:100, mouse, EMD Millipore), NRF2 (1:100, rabbit, Cell Signaling Technologies), or KEAP1 (1:100, mouse, Proteintech, Rosemont, IL, USA). For NRF2 and KEAP1 antibodies, cells were treated with 500 nM MG132 for 2 h before fixation to prevent the degradation of NRF2. NRF2 has a short half-life and is readily degraded in basal conditions. MG132 is a proteasome inhibitor and prevents the degradation of NRF2 and induces NRF2 nuclear translocation [41]. Coverslips were then washed for 5 min with PBS three times and for 1h at room temperature protected from light incubated with secondary antibody PBS and 10% normal donkey serum: Alexa Fluor 488 donkey anti-rabbit (1:100, Jackson Immuno Research, West Grove, PA, USA), CY3 donkey anti-rabbit (1:100, Jackson Immuno Research), CY3 donkey anti-mouse (1:100, Jackson Immuno Research), Alexa Fluor 488 donkey anti-mouse (1:100, Jackson Immuno Research), and DAPI (1:100, Molecular Probes, Eugene, OR, USA). The antibody solution was removed, and the coverslips were washed three times with PBS and mounted onto slides using Fluoroshield (Sigma-Aldrich). The Zeiss LM-800 confocal microscope was used to acquire fluorescent images. Image analysis was performed using ImageJ (National Institutes of Health, Bethesda, MD, USA, ImageJ 1.53t, https://imagej.net/ij/download.html. The corrected total cell fluorescence (CTCF) was calculated using the formula: CTCF = Integrated Density − (Area of selected cell x Mean fluorescence of background readings) [42]. For manual counting, 10 images were captured at random locations using the confocal microscope at 20× magnitude and the mean is determined. The total DAPI positive nuclei were counted using ImageJ.

### 2.8. Single Cell RNA-Sequencing Pipeline

NSCs were single cell dissociated with StemPro Accutase (Thermo Scientific) and resuspended in 3:2 NN1:C1 Suspension Reagent (3:2 ratio) to induce buoyancy in the cell suspension and provide efficient feeding into the microfluidic chambers of the C1 IFC chip (Fluidigm, South San Francisco, CA, USA). The cell suspension was loaded into a primed IFC chip for capturing. The IFC chip was prepared following Fluidigm’s protocol, which has been previously described in detail [43]. Single cell cDNA libraries were generated using SMART-Seq v4 chemistry (Clontech). Individual library concentrations were determined using the Qubit 3 fluorometer (Thermo Scientific). If the libraries did not fit within the concentrations of 100–300 pg/μL, they were diluted with C1 dilution reagent. The libraries were indexed using the Nextera XT DNA Library Preparation kit (Illumina, Foster City, CA, USA). Indexed libraries were pooled and fragment length was measured with the TapeStation 4200 (Agilent, Santa Clara, CA, USA). AMPure XP beads (Beckman Coulter, Brea, CA, USA) were used on the pooled libraries to isolate fragments 300–500 bp long. The pooled libraries were sequenced at 100 bp paired-end using the Illumina HiSeq 3000.

We previously reported the pipeline for single-cell RNA-seq bioinformatics analysis [44]. Briefly, raw data were quality checked using FASTQC. Sequences with PHRED score < 28 were removed from the analysis. Seventy-eight samples for Mut NSC and 67 samples for GC NSC passed QC. Using the human GRCh38.p10 reference genome from Ensembl (release 88), the paired sequences were pseudoaligned using kallisto (ver. 0.43.0) bootstrapped 100 times [45,46,47]. Sleuth was used downstream to quantify transcript abundances and filter out zero reads [48]. The R package SINGuLAR (ver. 3.6.2, Fluidigm) was used to perform differential expression analysis between the two cell lines with principal component analysis and unsupervised hierarchical clustering. The threshold of fold change was set to the default parameter of 2 and *p*-value was set to a default of <0.05. Functional annotation was performed using the DAVID Bioinformatics Resources [49,50] (ver. 6.7, Leidos Biomedical Research, Frederick, MD, USA). Ingenuity Pathway Analysis (IPA, QIAGEN, Redwood City, CA, USA) was used to identify overlapping canonical pathways.

### 2.9. Measuring Superoxide Content with MitoSOX Red Staining

MitoSOX Red (Thermo Scientific) is a fluorogenic dye, which targets mitochondria. Superoxides oxidize the reagent to produce a red fluorescence. Measurement of fluorescence is an indirect measurement of cellular superoxide production. MitoSOX Red staining was performed according to manufacturer’s instructions. NSCs and iPSCs were plated onto Geltrex coated coverslips for either 24 h for NSCs or 3 days for iPSCs and supplemented with either NSC culture media or mTeSR Plus media. The cells were incubated for 30 min with 2.5 μM of MitoSOX Red dye. Afterwards, the coverslips were fixed in 10% Formalin for 15 min. Coverslips were mounted onto glass microscope slides with Fluoroshield histology solution. Quantitative analysis was performed as described above in Section Immunocytochemistry. Fluorescent microscopic images were taken using Zeiss LSM-800 confocal microscope at random locations. Image analysis was performed using ImageJ. Corrected total cell fluorescence was calculated using the formula: CTCF = Integrated Density − (area of selected cell × Mean fluorescence of background readings).

### 2.10. Electron Paramagnetic Resonance Spectroscopy

NSCs were single cell dissociated and incubated for one hour at 37 °C in EPR buffer (Krebs Ringer buffer supplemented with 25 mM sodium bicarbonate, 20 mM HEPES, 5 μM silver diethyldithiocarbamate, and 25 μM deferoxamine) with spin probe 100 μM CMH (1-hydroxy-3-methoxycarbonyl 2,2,5,5-tetramethylpyrrolidine, Enzo Lifesciences, Farmingdale, NY, USA) or 25 μM MitoTEMPO (Enzo Lifesciences). For a positive control, 25 units superoxide dismutase (SOD, Sigma-Aldrich) or 25 μM of SOD mimetic (MnTBAP, Calbiochem, Saint Louis, MO, USA) were used. Cell suspension was collected and snap frozen. Once thawed, lysates were placed in either a capillary tube or a quartz flat cell. Signal intensity was measured using EMXnano bench-top EPR (Bruker, Billerica, MA, USA) using the following parameters: Receiver gain 40 dB, modulation amplitude 2.006 G, microwave attenuation 11 dB, center field 3427.30 G, sweep width 70 G, sweep time 26.24 s, and number of scans 1. Recordings were performed in triplicate. Signal intensity was normalized by sample protein concentration measured via BCA assay.

### 2.11. Measuring Cardiolipin Content

iPSCs were detached using 500 μM EDTA. Cells were counted using trypan blue and Countess auto-cell counter (Invitrogen). Cell suspension was diluted to 10 million cell density. The cells were homogenized in SHE buffer: 250 mM sucrose, 20 mM HEPES, 2 mM EGTA, 10 mM KCl, 1.5 mM MgCl_2_, and 0.1% defatted bovine serum albumin (BSA) supplemented with complete Minitab protease inhibitor cocktail (Sigma-Aldrich). For mitochondria isolation, the homogenate was centrifuged at 800× *g* for 2 min at 4 °C, the supernatant was recovered and centrifuged at 10,000× *g* for another 5 min to pellet mitochondria. The amount of cardiolipin present on the mitochondria was determined using a commercially available fluorometric assay kit (BioVision, Milpitas, CA, USA) according to manufacturer’s protocol. Mitochondria fraction was compared with cellular fraction. The fractions were stained with a cardiolipin-specific probe and quantified against a cardiolipin standard curve. Florescence was measured at Ex/Em 340/480 nm. Cardiolipin content was normalized with protein concentrations determined with BCA assay.

### 2.12. Measuring Lipid Peroxidation

Lipid peroxidation was measured using the Click-iT Lipid Peroxidation Detection with Linoleamide Alkyne kit (Thermo Scientific) following manufacturer’s protocol. NSCs were plated onto Geltrex coated coverslips supplemented with NSC NN1 culture media. Once confluent, 50 μM Click-iT LAA solution was added to the growth media and incubated for 2 h. The cells are then washed with PBS to remove free Click-iT LAA from the wells. Coverslips were then fixed in 4% paraformaldehyde for 15 min, washed with PBS and permeabilized with 0.5% Triton X-100 in PBS for 10 min at room temperature. Coverslips were blocked with 1% BSA in PBS for 30 min at room temperature. Blocking was removed and coverslips were washed with PBS. Click-iT reaction cocktail was added to each coverslip and incubated for 30 min at room temperature and protected from light. The reaction cocktail was removed, and the coverslips were washed with 1% BSA in PBS and then with PBS only. The coverslips were also counterstained with DAPI. Fluorescent microscopic images were taken using Zeiss LSM-800 confocal microscope at random locations. Image analysis was performed using ImageJ where both fluorescence and DAPI positive cells were counted. Corrected total cell fluorescence was calculated using the formula: CTCF = Integrated Density − (area of selected cell x Mean fluorescence of background readings). CTCF was normalized for DAPI+ count.

### 2.13. Seahorse Extracellular Flux Analyzer: Mito Stress Assay

The Extracellular Flux Analyzer XF96 (Agilent) was used to analyze cellular oxygen consumption and ATP production rate. NSCs were plated onto Geltrex coated XF96 microplates in octuplicates at 6.5 × 10^4^ cells/well, supplemented with NSC NN1 culture media. Rates of mitochondrial respiration and ATP production were measured via oxygen consumption rate (OCR, pmol/min) and extracellular acidification rate (ECAR, mPh/min). Oligomycin, FCCP, and Rotenone/Antimycin A were used at 1 μM concentration to induce fluctuations in OCR and ECAR. XF assay media supplemented with 5.5 mM glucose, 2 mM L-glutamine, and 1 mM sodium pyruvate. NSC DNA content was quantified using CyQUANT (Thermo Scientific) after completion of Mito Stress assay. Media was removed from each well of the microplate. The cells were frozen at −80 °C. The plate was thawed at room temperature and CyQUANT GR dye/cell lysis buffer was added to each sample well and incubated for 5 min at room temperature in the dark. Fluorescence was measured using a microplate reader set for 480 nm excitation and 520 nm emission maxima. OCR and ECAR recordings were normalized with the resultant DNA quantification. Data were processed using manufacturer’s calculation matrix to determine basal respiration level, proton leak, ATP production, maximal respiration, spare respiratory capacity, and non-mitochondrial oxygen consumption.

### 2.14. Measuring Intracellular Glutathione Concentration

Glutathione was measured using Glutathione Fluorometric Assay Kit (BioVision) following the manufacturer’s protocol. 4 × 10^6^ NSCs were homogenized in Glutathione Assay Buffer. Homogenate was mixed with perchloric acid, vortexed, and incubated on ice for 5 min. Homogenate was centrifuged for 2 min at 13,000 *g* at 4 °C and the supernatant, containing glutathione, was collected. 6N KOH was added to each sample to precipitate and neutralize perchloric acid. To detect total glutathione, a reducing agent mix was added to each sample to convert GSSG to GSH. To detect GSSG, GSH quencher was added to quench GSH, followed by the reducing agent mix that converts the GSSG to GSH. Finally, o-phthalaldehyde probe was added and samples were incubated at room temperature for 40 min. Samples were then read with a fluorescence plate reader equipped with EX/EM = 340/420 nm. Glutathione concentrations were quantified against a GSH standard and using the formula: Glutathione Concentration = Glutathione amount from standard curve/original sample volume added to sample wells.

### 2.15. Statistical Analysis

Statistical analysis was conducted using GraphPad Prism 9.0.0 software Inc (La Jolla, CA, USA). Significance in differences between two groups was performed by applying Student’s *t*-test where appropriate. For comparison of multiple groups, one-way analysis of variance (ANOVA) with Tukey’s post-hoc analysis was performed to identify the significant differences. A *p*-value of less than 0.05 was considered statistically significant.

## 3. Results

### 3.1. Characterization of iPSCs Derived from G2019S LRRK2 PD Patient

**Figure 1:**
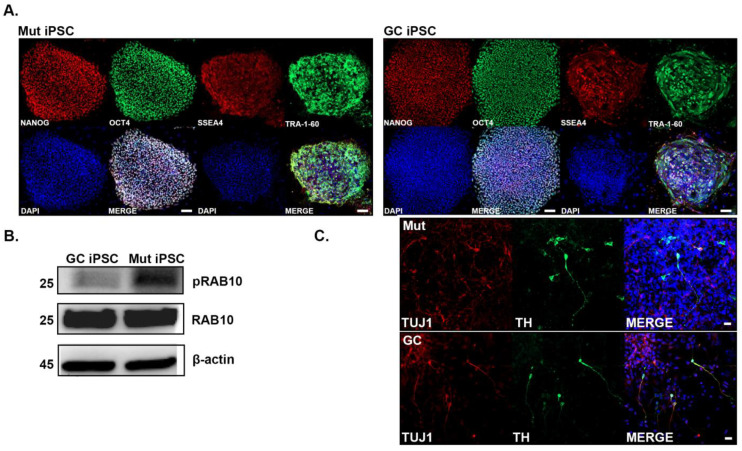
Characterization of *LRRK2* G2019S (Mut) iPSCs and isogenic gene corrected (GC) iPSCs. (**A**) Representative confocal images of Mut (Left) and GC (Right) iPSCs showing the expression of the pluripotent stem cell markers: NANOG (Red), Octamer-binding protein 4 (OCT4, Green), Stage-specific embryo antigen 4 (SSEA4, Red), and TRA-1-60 (Green). (**B**) Western blot analysis of lysates from Mut and GC iPSCs for LRRK2 kinase activity measured through levels of phosphorylation of RAB10 at T73. The cell lysates were also blotted to determine the change in total amount of RAB10 protein levels. β-actin was used as loading control. The molecular weights in kDa are represented on the right side of the panel. (**C**) Representative confocal images of Mut (Top) and GC (Bottom) showing similarities of iPSC-derived dopaminergic neurons in the two cell lines. ß-tubulin class III (TUJ1, Red) and Tyrosine hydroxylase (TH, Green). Scale bars are: 100 µm in (**A**), 20 µm in (**C**).

The iPSCs were generated from fibroblasts of a PD patient with heterozygous G2019S mutation (Mut iPSCs) and the isogenic gene corrected (GC iPSCs) lines were generated using zinc finger nuclease [37,38]. The iPSCs were characterized for pluripotency using pluripotent stem cell markers NANOG, OCT4, SSEA4, and TRA-1-60 (Figure 1A). Both Mut and GC iPSCs were positive for all four markers. Western blot analysis was performed on Mut and GC iPSC lysates to confirm the LRRK2 kinase activity. We measured the levels of RAB10 because it is a well-established target of LRRK2 phosphorylation [51] and thus a marker of LRRK2 kinase activity. We observed an increase in levels of pRAB10 in Mut iPSCs compared with GC iPSCs (Figure 1B). There was no difference observed in the total amount of RAB10 indicating that there is greater LRRK2 kinase activity in Mut iPSCs than GC iPSCs. NSCs were derived from the iPSCs and propagated in suspension as neurospheres [39] (Figure 1C). NSCs were then differentiated into neurons and dopaminergic neurons as we previously reported (Figure 1C) [40].

### 3.2. Single-Cell Transcriptomic Analysis of Mut iPSCs and GC iPSCs Revealed Defect in the NRF2-Mediated Oxidative Stress Response among Other Pathways

**Figure 2:**
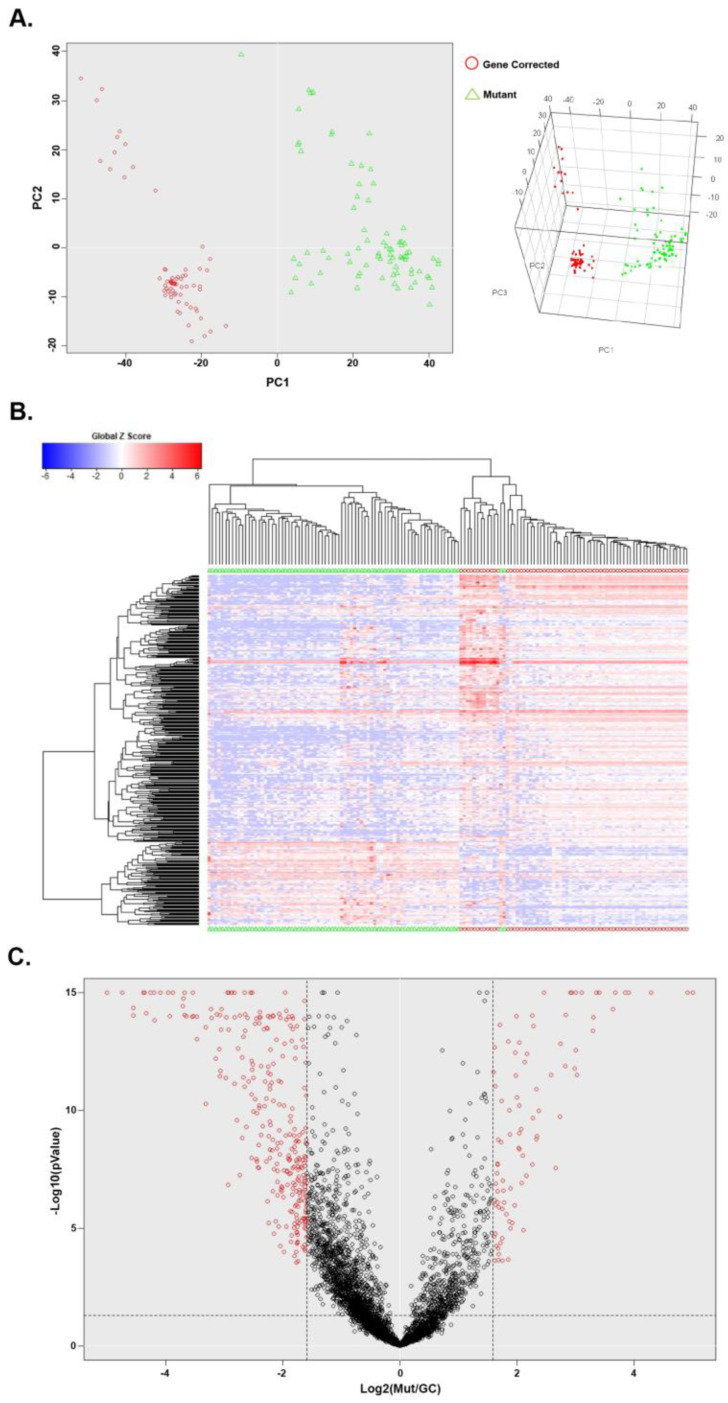
Single-cell RNA-seq reveals significant differentially expressed genes between the Mut and isogenic GC iPSCs. Single-cell RNA-seq was performed to compare GC NSC (*n* = 67) and Mut NSC (*n* = 78). (**A**) Principal component analysis (PCA) shows distinct clustering of GC (Red Circle) and Mut (Green Triangle) NSCs. (**B**) Heatmap showing expression levels of all genes after performing hierarchical clustering. (**C**) Volcano plot reveals 91 upregulated genes and 292 downregulated genes where fold change threshold is set at 2 and *p* ≤ 0.05.

To determine the impact of the G2019S *LRRK2* mutation on the cellular metabolic functions we performed single cell RNA-seq (scRNA-seq) transcriptomic profiling on the Mut iPSC line and on its isogenic gene corrected counterpart. Differentiated NSCs were single cell dissociated for scRNA-seq using the Fluidigm C1 pipeline. The mRNA of 96 individual cells of Mut NSCs and GC NSCs were sequenced, of which, 78 Mut NSCs and 67 GC NSCs passed the quality check with an average of about 4 million reads per sample and were aligned to the GRCh38.p10 reference genome. We then performed differential expression analysis on the aligned reads. Principal component analysis (PCA) confirmed distinct clustering of GC and Mut NSCs indicating significant variance between the two (Figure 2A). Unsupervised hierarchical clustering recapitulates this variance (Figure 2B). A total of 2363 genes were annotated within our data set, of which 292 genes were significantly downregulated and 91 genes were significantly upregulated with a threshold fold change greater than or equal to 2 (Figure 2C).

**Figure 3:**
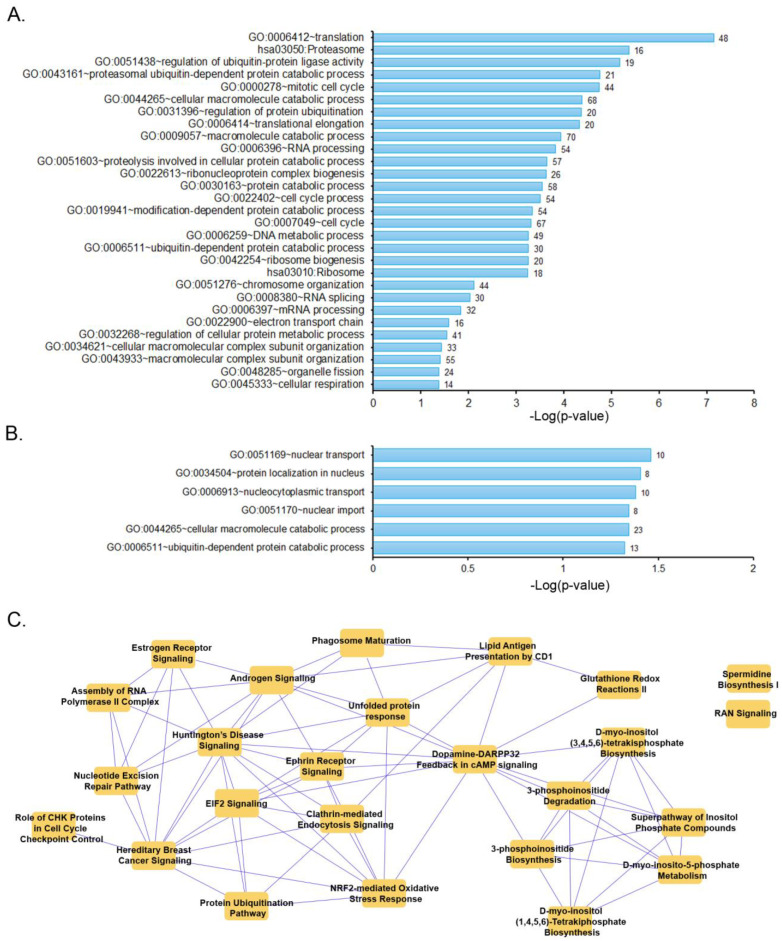
Pathway analysis of significant differentially expressed genes. Gene ontology (GO) term enrichment analysis using DAVID functional annotation (**A**,**B**). Gene set of all significantly downregulated genes (**A**) and significantly downregulated genes (**B**). Gene ontology was selected based on *p* ≤ 0.05. (**C**) Overlapping canonical pathways identified using Ingenuity Pathway Analysis (IPA) of all significantly downregulated genes.

A pathway analysis was then performed with the list of significantly different genes. Functional annotation of downregulated genes showed enrichment for translation, regulation of ubiquitin-mediated proteolysis, electron transport chain, organelle fission, and cellular respiration (Figure 3A). On the other hand, functional annotation of upregulated genes showed particular enrichment for nuclear transport (Figure 3B). Further analysis was then performed using Ingenuity Pathway Analysis (IPA), which revealed additional overlapping canonical pathways. Notably, we identified pathways involved in the clathrin-mediated endocytosis, unfolded protein response, NRF2-mediated oxidative stress response, and glutathione redox reactions (Figure 3C).

### 3.3. LRRK2 G2019S Cells Exhibit Increased Basal Oxidative Stress

**Figure 4:**
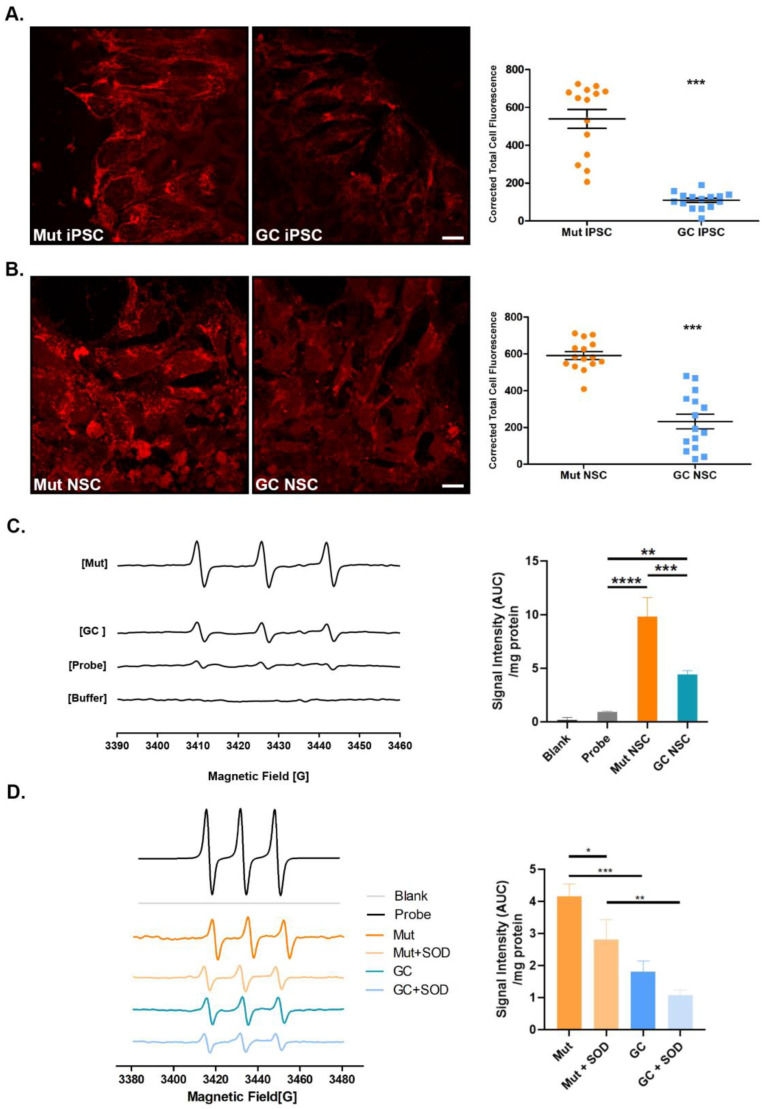
*LRRK2* Mut iPSCs and NSCs exhibit increased levels of basal intracellular ROS. Mitosox Red staining of Mut and GC iPSCs and NSCs (**A**,**B**). Representative confocal images of Mut and GC iPSCs (**A**) and Mut and GC NSCs (**B**). Corrected total cell fluorescence (CTCF) was measured with ImageJ as described in Method section. Data represent mean ± SEM of experiments performed two or three times on independent culture preparations, each performed in duplicate or triplicate. *** *p* ≤ 0.001. (**C**) Electron paramagnetic (EPR) spectroscopy using 1-hydroxy-3-methoxycarbonyl-2,2,5,5-tetramethylpyrrolidine (CMH) probe to measure superoxide content in Mut (Orange) and GC (Blue) NSCs. Recordings were conducted with borosilicate capillary tubes. (**D**) EPR using MitoTEMPO probe to measure antioxidant potential in Mut (Orange) and GC (Blue) NSCs. Recordings were conducted with a quartz flat cell. Signal intensity was determined by calculating area under the curve of the EPR spectrum (Left). Signal intensity was normalized by protein concentration. Recordings were conducted in triplicate. Data represent mean ± s.e.m. * *p* ≤ 0.05, ** *p* ≤ 0.01, *** *p* ≤ 0.001 and **** *p* ≤ 0.0001. Scale bars are 10 µm in (**A**,**B**).

Our single cell transcriptomics confirmed the implication of oxidative stress in the G2019S LRRK2 pathologies. We then sought to investigate whether mitochondria function was affected in the G2019S versus its isogenic gene corrected counterpart. MitoSOX Red is a fluorogenic dye that targets mitochondria and is used to label intracellular superoxides. Superoxides oxidize the reagent to produce a red fluorescence. Measurement of fluorescence is an indirect measurement of cellular superoxide production. There was a significant increase in MitoSOX Red fluorescence intensity in both Mut iPSCs and NSCs compared with GC iPSCs and NSCs (Figure 4A,B). This suggests that there is a greater amount of superoxide in the mutant cells in basal conditions. Furthermore, the morphology of the mitochondria appears to be more punctate (Figure 4A,B), suggestive of mitochondrial fission most likely caused by oxidative stress [52].

To corroborate our findings with the MitoSOX Red staining, we used electron paramagnetic resonance spectroscopy (EPR). EPR is a method used to detect unpaired electrons or free radicals to identify, quantify, and visualize short-lived reactive oxygen species (ROS). A variety of free radicals can be detected when utilizing different spin probes. The CMH (1-hydroxy-3-methoxycarbonyl 2,2,5,5-tetramethylpyrrolidine) probe was used to directly measure intracellular production of superoxide ion, peroxyl radical, peroxynitrite, and nitrogen dioxides. The oxidation of CMH produces 3-methyoxycarbonyl-proxyl nitroxide, which is detected by the benchtop EPR. When the probe is oxidized, the EPR records higher peaks on the spectra; ergo larger peaks indicate more intracellular ROS. NSCs were derived from the Mut and GC iPSCs and single cell dissociated into EPR buffer and CMH probe. Signal intensities were determined by calculating the area under the curve of the EPR spectra and taking the second integral of the curve. The signal intensity increased when CMH probe was incubated with Mut NSCs compared with GC NSCs (Figure 4C), indicating levels of superoxides were significantly higher in Mut NSCs than in GC NSCs. Next, we measured the antioxidant potential of the cells using MitoTEMPO. MitoTEMPO is a mitochondria-targeting superoxide dismutase mimetic that scavenges superoxide and alkyl radicals. It has a lipophilic cation triphenylphosphonium allowing it to circumvent the lipid bilayer and accumulate in the mitochondria. The EPR spectra from MitoTEMPO is opposite from that of CMH probe, where lower signal intensity indicates greater antioxidant potential. With the MitoTEMPO probe, we also observed a higher signal intensity in the Mut NSCs, suggesting a reduced antioxidant response (Figure 4D). To verify that the signal intensity would be reduced with antioxidants, we also incubated our samples with superoxide dismutase (SOD) and MnTBAP (SOD mimetic). We saw a significant reduction in signal intensity when Mut NSCs were treated with either SOD or MnTBAP (SOD mimetic), verifying that the greater signal intensity was due to a perturbed antioxidant response.

### 3.4. Mitochondrial Deficits in G2019S LRRK2 Cells

**Figure 5:**
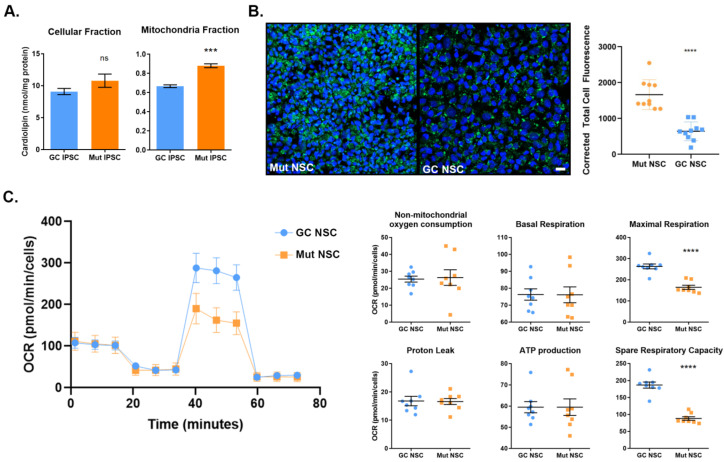
*LRRK2* Mut iPSCs and NSCs exhibit increased mitochondrial lipid peroxidation. (**A**) Cardiolipin concentration was measured in the isolated cellular fraction and the mitochondria fraction of GC (Blue) and Mut (Orange) iPSCs. (**B**) Confocal photomicrograph of Mut and GC iPSCs showing the lipid peroxidation of Mut vs. GC NSCs. During oxidation, the modified linoleic acid produces alkyne-modified protein that is multiplexed with Alexa Fluor 488 to produce a green fluorescence indicative of lipid peroxidation. The lipid peroxidation of Mut and GC NSCs was measured using confocal microscopy and the corrected total cell fluorescence (CTCF) measured with ImageJ. (**C**) Mitochondrial respiration of GC and Mut NSCs was measured using Seahorse. See Results section for description. Data represent mean ± SEM of experiments performed two or three times on independent culture preparations, each performed in duplicate or triplicate. *** *p* ≤ 0.001, **** *p* ≤ 0.0001. Scale bar is 20 µm in (**B**).

We next wanted to confirm whether mitochondrial deficits that were observed in our MitoSOX staining assay where Mut cells showed LRRK2-induced mitochondrial fragmentation. Cardiolipin is biosynthesized in the inner mitochondrial membrane, where it plays an important role. It is required to maintain structure and enzymatic activity of the electron transport chain. Also, it serves as a proton trap for oxidative phosphorylation, minimizing pH changes within the inner membrane. Cardiolipin plays an important role in mitochondrial protein import and can initiate apoptosis when it becomes oxidized and distributed to the outer mitochondrial membrane. Cardiolipin content can be measured with a fluorometric probe. Changes in the amount of cardiolipin may suggest an oxidative stress response as it is susceptible to ROS. Oxidative damage to cardiolipin negatively impacts its function in the mitochondrial membrane by altering membrane dynamics and reducing oxidative phosphorylation efficiency, leading to apoptosis [53]. We isolated mitochondria from Mut and GC iPSCs and compared it with whole cell suspensions to determine whether the differences in cardiolipin content is observed only in the mitochondria. We observed a significant increase in mitochondrial cardiolipin content only in Mut iPSCs (Figure 5A). The data suggest that the increase in cardiolipin may be due to a response against increased ROS.

Since we observed a difference in the amount of cardiolipin in Mut iPSCs, we asked the question whether this was also accompanied with an increase in lipid peroxidation. Lipid peroxidation can be visualized using Click-iT Lipid Peroxidation detection with linoleamide alkyne. This kit utilizes an alkyne-modified linoleic acid that incorporates into cellular membranes. When the linoleic acid is oxidized during lipid peroxidation, it produces 9- and 13-hydroperoxy-octadecadienoic acid (HPODE). In turn, the hydroperoxides decompose to α,β-unsaturated aldehydes that then modify proteins at nucleophilic side chains. With copper-catalyzed click chemistry, the alkyne-modified proteins are multiplexed with a fluorophore that can be imaged with fluorescence microscopy. Indeed, we observed a significantly higher fluorescence in Mut NSCs indicating increased lipid peroxidation (Figure 5B).

As both cardiolipin content and lipid peroxidation were increased in *LRRK2* Mut cells, we investigated whether there was an effect on mitochondrial function. We performed the Mito Stress Assay on the Seahorse Extracellular Flux Analyzer with Mut and GC NSCs. The Seahorse measures oxygen consumption rate. Oligomycin was used to determine the proportion of ATP-linked ACR and FCCP, a potent uncoupler that induces maximal respiration by inducing proton transport across the inner membrane. Mut NSCs showed a significantly decreased maximal respiration compared with GC NSCs (Figure 5C). Spare respiratory capacity, the capacity of a cell to respond to energetic demands, was determined by the difference between maximal respiration and basal respiration. Mut NSCs also showed a significant decrease in spare respiratory capacity. There was no statistical difference between Mut and GC NSCs in the context of basal respiration, ATP production, proton leakage, or non-mitochondrial oxygen consumption.

### 3.5. Antioxidant Response Is Perturbed in G2019S LRRK2 Cells

**Figure 6:**
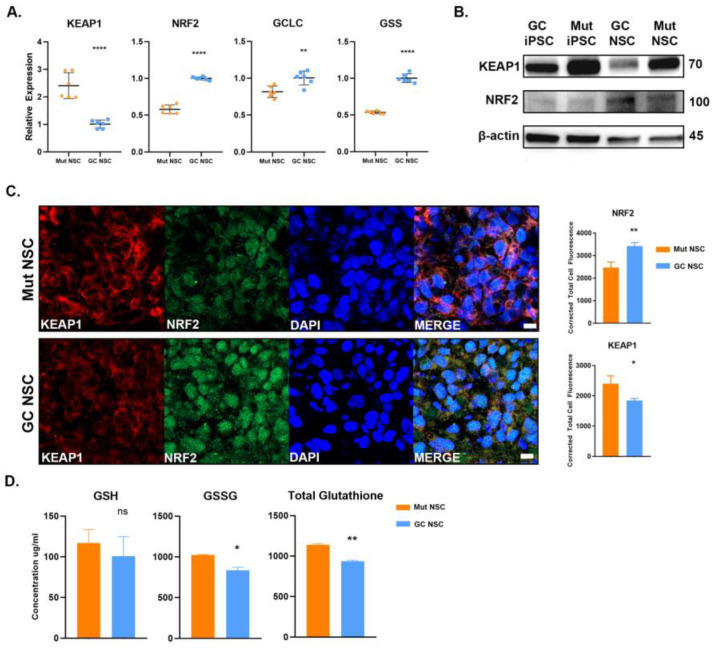
*LRRK2* Mut NSCs exhibit reduced antioxidant response. (**A**) Gene expression for KEAP1, NRF2, GCLC, and GSS of Mut (Orange) and GC (Blue) NSCs were measured with qPCR performed in triplicates in two independent experiments. (**B**) Western blot analysis of lysates from Mut and GC iPSCs and NSCs labeled for KEAP1 and NRF2 protein levels. β-actin used as loading control. The molecular weights in kDa are represented on the left side of the panel. (**C**) Representative confocal images of Mut and GC NSCs stained for KEAP1 (RED) and NRF2 (Green). Cells were treated with 500 nM MG132 for 2 h prior to staining to prevent the degradation of NRF2. Corrected total cell fluorescence (CTCF) was measured with ImageJ. (**D**) Glutathione concentration of Mut (Orange) and GC (Blue) NSCs were measured with a fluorometric assay. Data represent mean ± SEM of experiments performed two or three times on independent culture preparations, each performed in duplicate or triplicate. * *p* ≤ 0.05, ** *p* ≤ 0.01, and **** *p* ≤ 0.0001. Scale bar is 10 µm in (**C**).

The G2019S mutant cells exhibited increased basal ROS that was potentially due to a perturbed antioxidant response, we therefore set out to determine if there was a lack of antioxidant synthesis. Nuclear factor E2-related factor 2 (NRF2) regulates the expression of antioxidant response elements in response to oxidative stress. Under normal conditions, NRF2 is localized in the cytoplasm and bound to Kelch like-ECH-associated protein 1 (KEAP1) and ubiquitinated for degradation. In response to oxidative stress, KEAP1 releases NRF2, which then translocates into the nucleus to transcribe antioxidant response elements, such as glutathione S-transferase, Thioredoxin reductase 1, and glutamate-cysteine ligase (GCL). Dysfunction in the antioxidant response may lead to accumulation of free radical species. We measured the gene expression of KEAP1, NRF2, Glutamate-cysteine ligase (GCLC) and glutathione synthetase (GSS) (Figure 6A). We observed that there was a significant increase in expression for KEAP1 in Mut NSCs compared with GC NSCs. Furthermore, we observed a significant decrease in NRF2, GSS, and GCLC expression in Mut NSCs suggesting a reduced antioxidant response. Western blot analysis confirmed that KEAP1 protein levels were higher in Mut NSCs (Figure 6B). Immunocytochemistry and quantitative analysis demonstrated a reduction in NRF2 in Mut iPSCs vs. the GC iPSCs (Figure 6C). It was also evident that there was a reduction in NRF2 translocation into the nucleus in Mut iPSCs. These data provide further evidence of a reduced antioxidant response.

We then asked the question whether increased levels of KEAP1 would have an effect on the antioxidant production. Glutathione is a tripeptide made up of glutamate, cysteine, and glycine. It exists as reduced (GSH) and oxidized (GSSG). GSH is one of the most abundant and powerful intracellular antioxidants. We measured GSH, GSSG, and total glutathione content of Mut and GC NSCs using a fluorometric assay. Interestingly, we observed no significant difference in reduced glutathione (GSH) concentration (Figure 6D). Although it appeared that the Mut NSCs had a significantly greater concentration of total glutathione, which may suggest that there was not a lack of antioxidant production. However, GSSG made up the bulk of the total glutathione which suggests that the increased ROS observed in Mut NSCs contributes to the significant increase in GSSG.

### 3.6. Antioxidant Effects of PRC-210 and Edaravone on the LRRK2 Compromised Oxidative Stress Response

**Figure 7:**
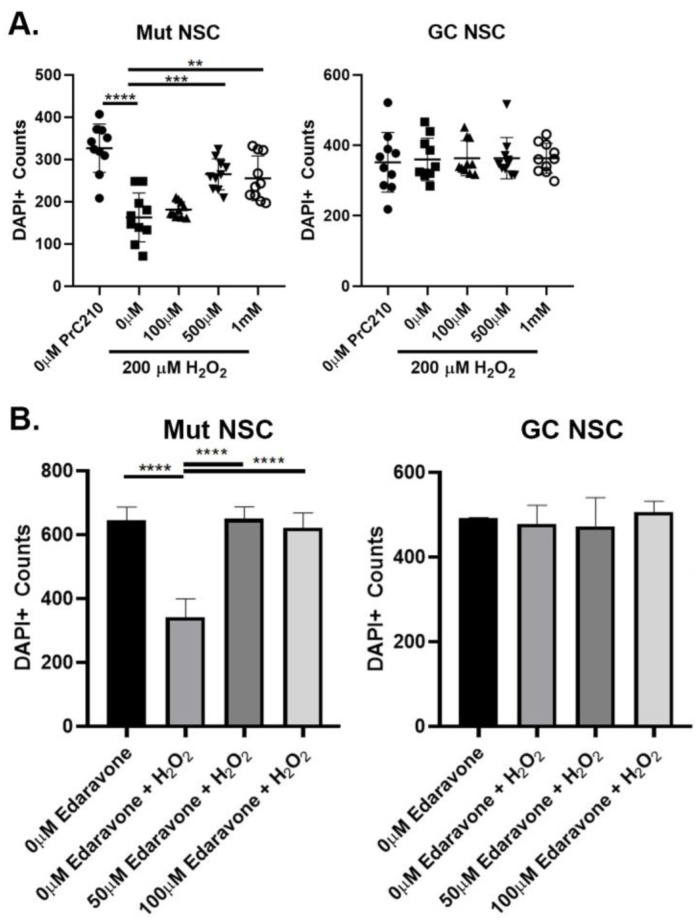
Antioxidant neuroprotective effects of PRC-210 and Edaravone. Cell viability in the H_2_O_2_-induced oxidative stress assay was measured through quantification of DAPI+ cells. (**A**) Mut (Left) and GC (Right) NSCs were treated with 200 µM H_2_O_2_ for 2 h combined with increasing concentrations of the antioxidant PrC-210 at 0 µM, 100 µM, 500 µM and 1 mM concentrations. (**B**) Mut NSCs and GC NSCs were treated with 200 µM H_2_O_2_ for 2 h combined with increasing concentrations of the antioxidant Edaravone at concentrations 50 µM and 100 µM. DAPI+ cells were manually counted using ImageJ. Data represent mean ± SEM of experiments performed two or three times on independent culture preparations, each performed in duplicate or triplicate. ** *p* ≤ 0.01, *** *p* ≤ 0.001, and **** *p* ≤ 0.0001.

It has been previously reported that G2019S *LRRK2* cells are more susceptible to oxidative stress [15,54]. To test the antioxidant effects of two potent antioxidant drugs, PrC-210 [55] and Edaravone, we used the hydrogen peroxide (H_2_O_2_)-induced oxidative stress assay. To determine the effective doses of the two compounds, NSCs were exposed to 200 µM hydrogen peroxide (H_2_O_2_) and to increasing concentrations of PrC-210 or Edaravone (Figure 7). The data show that Mut NSCs have decreased viability after H_2_O_2_ insult and that the cell viability was rescued with PrC-210 at concentrations of 500 µM and 1 mM (Figure 7A) and with Edaravone at concentrations 50 µM and 100 µM (Figure 7A,B).

## 4. Discussion

We used single cell transcriptomic analysis of familial PD patient-derived iPSC and its gene-edited isogenic control to identify cellular pathways involved in the pathogenic G2019S *LRRK2* mutation. The mutant cells showed defective oxidative response, unfolded protein response, ubiquitin proteolysis, and cellular respiration. Specifically, the G2019S *LRRK2* mutation caused an increase in superoxide production causing mitochondrial fission and a reduced antioxidant response. There was an increase in cardiolipin content and lipid peroxidation in the *LRRK2* mutant cells associated with a significant decrease in spare respiratory capacity. We report the involvement of the NRF2—KEAP1 pathway in the antioxidant response. There was a significant increase in expression for KEAP1 in Mut NSCs compared with GC NSCs concomitant with a significant decrease in NRF2, GSS, and GCLC expression in Mut NSCs suggesting a reduced antioxidant response. Finally, we tested the efficacy of two antioxidant compounds, PrC-210 and Edaravone in the H_2_O_2_ induced oxidative stress assay. We report a dose response protective effect in the Mut NSCs.

The mutant iPSC derived NSC progeny exhibited higher levels of ROS compared with the isogenic control. We observed greater fluorescence of MitoSOX red staining in *LRRK2* mutant cells, indicating higher concentrations of mitochondrial superoxides. Consistent with previous studies, we observed an increase in mitochondrial fragmentation in Mutant iPSCs and NSCs [56,57]. Furthermore, we observed increased cardiolipin content and subsequent lipid peroxidation. In turn, we observed that Mut NSCs exhibited reduced maximal respiration and spare respiratory capacity. It has been previously shown that NSCs derived from iPSCs harboring the G2019S *LRRK2* mutation displayed reduced maximal respiration capacity compared with wildtype [57]. This reduction in oxidative phosphorylation efficiency may be due to increased peroxidation of inner mitochondrial membrane cardiolipin, destabilizing the electron transport chain. These observations are consistent with our scRNA-seq data, which demonstrated a reduction in the expression of genes related to translational regulation of ubiquitin-mediated proteolysis, electron transport chain, organelle fission, and cellular respiration.

It has been hypothesized that the elevated ROS levels observed in the G2019S mutation contribute to increased cell death [15,54]. These data are consistent with our findings, we observed that *LRRK2* mutant cells are more susceptible to ROS and that antioxidants were effective in reducing ROS induced cell death. Indeed, antioxidant therapy has been investigated to prevent neurodegeneration [58,59]. Previous reports demonstrated that antioxidants sequester ROS and protect against mitochondrial damage [60].

NRF2 is the master regulator of the antioxidant response and drives the transcription of antioxidant response elements. We demonstrated that G2019S *LRRK2* affects the transcription of NRF2, KEAP1, and antioxidant response elements. We observed that there was a perturbed antioxidant response in *LRRK2* mutant cells. However, when we measured levels of glutathione, we found no difference in the amount of GSH. Previous reports have shown that there is a significant reduction in GSH in PD postmortem substantia nigra [61,62]. However, there was no difference in the total amount of glutathione in other regions of the brain. This lack of difference between the Mut and GC NSCs may be due to cell type specificity that may contribute to the reduction in GSH. During oxidative stress, GSSG is transported out of the cell via membrane y-GTP to prevent toxic effects to cellular function [63,64] leading to cell death [65]. The contribution of the elevated GSSG levels in G2019S mutant cells towards PD pathology remains to be explored.

## 5. Conclusions

**Figure 8:**
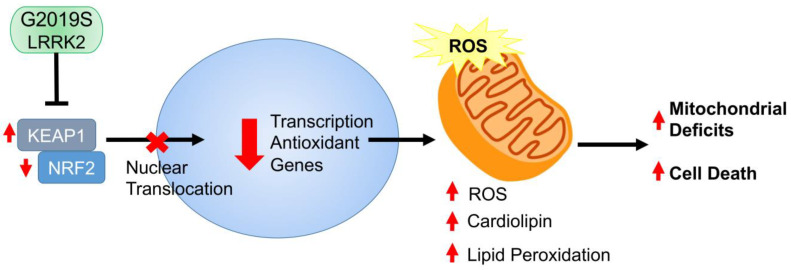
*LRRK2* G2019S reduces antioxidant response. Our data suggest that the hyperactive LRRK2 G2019S kinase activity leads to increase in KEAP1, which binds NRF2 and leads to its degradation, reduction in the antioxidant response, increased ROS, mitochondria dysfunction, and cell death.

The data presented here suggest that G2019S *LRRK2* mutation attenuates the antioxidant response through the NRF2-KEAP1 pathway (Figure 8) causing the cells to be susceptible to oxidative stress and cell death. Our data also highlights the relevance of single cell multiome analysis of iPSCs and their isogenic control in modeling familial PD harboring *LRRK2* mutations. Further studies are needed, and our goal is to elucidate the role of the NRF2-KEAP1 pathway in cell type-specific *LRRK2* induced pathogenesis in PD.

## Data Availability

Requests for information and protocols should be directed to the corresponding author.
